# The complete plastome genome and its inference on the phylogenetic position of *Bletilla formosana* (Orchidaceae)

**DOI:** 10.1080/23802359.2019.1688699

**Published:** 2019-11-13

**Authors:** Xiayu Wu, Cuixian Peng, Zhimin Li, Shaotian Chen

**Affiliations:** a School of Life Sciences, Yunnan Normal University, Kunming, China;; b Wenshan Academy of Agricultural Sciences, Wenshan, China;; c College of Pharmaceutical Science, Yunnan University of Chinese Medicine, Kunming, China

**Keywords:** Chloroplast genome, *Bletilla formosana*, orchid, overexploitation

## Abstract

*Bletilla formosana* is a traditional Chinese medicinal herb and recently threatened by overexploitation owing to the increasing demand. In present study, we sequenced and assembled the complete chloroplast (cp) genomes of this species. The plastome genome is a typical quadripartite circle molecule with the total lenth of 158,968 bp and overall GC content of 37.3%. We predicted 104 genes in the cp genome, including 80 protein coding gens, 20 tRNA and 4 rRNA. Phylogenetic analysis shows that *B*. *formosana* is sister to *B*. *striata*.

*Bletilla formosana* (Hayata) Schlechter is a traditional Chinese medicinal herb (Peng and Xiang 2014), and it is the most widely distributed member of the genus *Bletilla* Rchb. F., and the species is found in nine provinces of China (SE Gansu, Guangxi, Guizhou, Jiangxi, S Shaanxi, Sichuan, Taiwan, SE Xizang, Yunnan, and also in Japan (Ryukyu Islands) (Chen et al. 2009). But recently, this species is rarely found in nature, because the demand picked up and it has been over-exploited. In this paper, we presented the structure and characters of the chloroplast genome of *B. formosana*, which will facilitate the further researches on the phylogeny of the genus and conservation genetics of the species.

We collected the sample of *Bletilla formosana* from Jindun, Heqing County, Yunnan Province (100°11′54.92″E, 26°29′53.42″N), and deposited the voucher (C13012) in the Museum of Ethnic Medicine, Yunnan University of Chinese Medicine. The total genomic DNA was isolated using a modified CTAB method (Doyle and Doyle 1987), and the chloroplast genome was amplified using nine universal primer pairs following the recommended protocol (Yang et al. 2014). The amplification product was sequenced on the Illumina Hiseq 2000 platform. Finally, the plastome genome was assembled and annotated using Geneious 8.1 (Kearse et al. 2012), with reference to *B*. *striata* (Thunb. Ex A. Murray) Rchb. F. (GenBank Accession no.: KT588924). The complete plastid genome of *B*. *formosana* is a circular DNA molecule of 158,968 bp in total with a typical quadripartite structure, and its overall GC content was 37.3%. The plastome contains a large single copy (LSC) region of 86,824 bp and a short single copy (SSC) region of 18,718 bp, which are linked by two inverted repeat (IR) regions of 26,713 bp, and their GC content values are 35.1%, 30.4% and 43.2%, respectively. The cp genome is composed of 104 genes, which are 80 protein coding genes, 20 tRNA genes and 4 rRNA genes. The new annotated complete chloroplast genome was submitted to GenBank with accession number MN526744.

To further investigate the phylogenetic position of *Bletilla formosana*, we selected complete chloroplast genome sequences of 14 Orchidaceae species including *B*. *formosana*, and *Cephalanthera longifolia* (Linn.) Fritsch was assigned as the outgroup. These sequences were aligned using Geneious 8.1 (Kearse et al. 2012) and a neighbor-joining (Saitou and Nei 1987) tree was constructed using MEGA7 (Kumar et al. 2016), with 1000 bootstrap replicates. Our results show that three species of the genus *Bletilla* constitute a monophyletic group, and *B*. *formosana* is sister to *B*. *striata* (Figure 1), which is not consist with other study based on amino acid sequences of 39 protein coding genes from chloroplast genomes (Feng et al. 2019).

**Figure 1.**
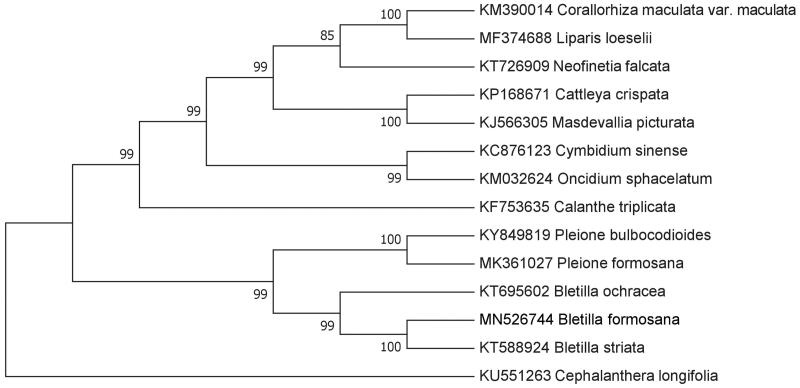
Phylogenetic position of *Bletilla formosana* inferred from 14 chloroplast genomes. Bootstrap support is indicated for each node.
